# A Theoretical Model to Investigate the Influence of Temperature, Reactions of the Population and the Government on the COVID-19 Outbreak in Turkey

**DOI:** 10.1017/dmp.2020.322

**Published:** 2020-09-09

**Authors:** Yahya Öz

**Affiliations:** Turkish Aerospace, R&D and Prototype Operations Directorate, Department of Innovation, Advanced Materials, Processes and Energies Technology Center, Ankara, Turkey

**Keywords:** coronavirus ınfections, COVID-19, pandemics, severe acute respiratory syndrome coronavirus 2

## Abstract

**Objectives:**

The ongoing coronavirus disease 2019 (COVID-19) pandemic, which was initially identified in December 2019 in the city of Wuhan in China, poses a major threat to worldwide health care. By August 04, 2020, there were globally 695,848 deaths (Johns Hopkins University, https://coronavirus.jhu.edu/map.html). A total of 5765 of them come from Turkey (Johns Hopkins University, https://coronavirus.jhu.edu/map.html). As a result, various governments and their respective populations have taken strong measures to control the spread of the pandemic. In this study, a model that is by construction able to describe both government actions and individual reactions in addition to the well-known exponential spread is presented. Moreover, the influence of the weather is included. This approach demonstrates a quantitative method to track these dynamic influences. This makes it possible to numerically estimate the influence that various private or state measures that were put into effect to contain the pandemic had at time *t*. This might serve governments across the world by allowing them to plan their actions based on quantitative data to minimize the social and economic consequences of their containment strategies.

**Methods:**

A compartmental model based on SEIR that includes the risk perception of the population by an additional differential equation and uses an implicit time-dependent transmission rate is constructed. Within this model, the transmission rate depends on temperature, population, and government actions, which in turn depend on time. The model was tested using different scenarios, with the different dynamic influences being mathematically switched on and off. In addition, the real data of infected coronavirus cases in Turkey were compared with the results of the model.

**Results:**

The mathematical study of the influence of the different parameters is presented through different scenarios. Remarkably, the last scenario is also an example of a theoretical mitigation strategy that shows its maximum in August 2020. In addition, the results of the model are compared with the real data from Turkey using conventional fitting that shows good agreement.

**Conclusions:**

Although most countries activated their pandemic plans, significant disruptions in health-care systems occurred. The framework of this model seems to be valid for a numerical analysis of dynamic processes that occur during the COVID-19 outbreak due to weather and human reactions. As a result, the effects of the measures introduced could be better planned in advance by use of this model.

A total of 18,364,694^1^ confirmed cases of the ongoing coronavirus disease 2019 (COVID-19) pandemic are known until August 04, 2020. There are 234,9341 only in Turkey. Compared with the other 2 epidemics in the 21st century caused by coronaviruses, the 2002-2004 severe acute respiratory syndrome (SARS) and 2012 Middle East respiratory syndrome (MERS) outbreaks with first confirmed cases in China and Saudi Arabia, respectively, the number of deaths is significantly higher.^2,3^ The first case in Turkey was reported on March 11, 2020.^1^ Since then, drastic measures have been taken to contain the spread of the virus. In addition to the closure of schools and universities, this includes, for example, the closure of restaurants, wearing surgical masks, travel restrictions, and curfew for certain age groups and all citizens for small times.^4^ Especially long-term interventions with low social and economic costs, such as aggressive testing and working from home in all company departments that can guarantee this, seem to be extremely beneficial and were implemented to a certain extent.^4^ In addition, economic aid packages were prepared to protect both industry and prevent social decline. Moreover, similar programs have been launched around the world.^5,6^ However, a severe impact of the COVID-19 outbreak is already measurable and a worldwide recession is expected.^7^

Of interest, there are parallels to the well-known 1918-1920 Spanish flu pandemic caused by an influenza virus. This pandemic was the deadliest in human history. Estimates assume that around one-third of humanity was infected and the number of deaths ranged from 50 to 100 million.^8^ Several waves of the outbreak occurred, with many regions shaken by up to 3 waves.^9-11^ Remarkably, pre-existing immunity within sections of the populations around the world might have been present.^12^ As a reaction to the pandemic, school closings were decided in certain countries and panic reactions of the people occured. The mildness of many disease courses, incubation times, and pneumonia^13-15^ as a possible result of both diseases are also quite similar. Furthermore, the fatality rate of 2% for the Spanish flu is in the same order of COVID-19. Hence, characteristics of the pandemics, Spanish flu and COVID-19, are comparable.

Models describing the 1918-1920 pandemic while taking weather and individual and government reactions into account have been published in the corresponding literature. Results show that high temperature and humidity led to a reduced virus survival.^16,17^ Moreover, government reactions in different regions that yielded a decreasing number of human contacts resulted in a temporal pattern of death rates.^18^ In combination with the individual responses this might have caused varying transmission rates that in turn might have led to the different waves of the disease.^19^ Note that the integration of individual reactions into epidemic models was accomplished by several different methods.^20,21^ In particular, mathematical tools such as Monte Carlo experiments and iterated filtering methods for nonlinear stochastic dynamical systems, that are typically used in various fields like mathematical physics, were further developed for applications such as the numerical analysis of pandemics.^22^ Additionally, modeling approaches that use compartmental models in epidemiology, especially Susceptible-Exposed-Infectious-Removed (SEIR) models, and other methodologies for COVID-19 were used.^23,24^

Hence, the motivation of this study is 2-fold. First, a model that combines the previous work for influenza and coronavirus is proposed. This model is conceptually able to not just describe the spread of the pandemic. Instead, the influence of seasonal effects as well as containment strategies of governments are included. Furthermore, variable individual reactions based on the risk perception is incorporated. Note that the emerging literature on COVID-19 includes the environmental perspective in accordance with this presented study. While a few studies conclude that varying temperatures have little or no influence on the spread of the virus,^25,26^ most studies report in contrast that rising humidity and temperatures lead to a decreasing transmission rate.^27-29^ However, wind speed is negatively correlated with the mean temperature and increases the transmission rate^25^ while rainfall seems not to be significantly correlated to COVID-19.^28^ Furthermore, it is well-known^28^ that a high population density leads to very fast transmission of COVID-19.

Second, this model might be used for the purpose of providing a projection for the development of the outbreak in Turkey for the coming months. Fitting the parameters to the real data shows that the model can represent them, which in turn means that the government can quantitatively assess their previous reactions at time *t*. The goal of this is to make a first step on paving a difficult but viable path that prevents the collapse of the health-care system of Turkey while minimizing social and economic effects as much as possible. Furthermore, the prevention of possible further waves of the pandemic by intervening quantitatively well-judged will be crucial to decrease the impact of the outbreak.

## METHODS

### Modified SEIR Model

A previously developed model^30^ for an influenza outbreak is adopted for the purposes of this study. The starting point of the proposed model is the SEIR model. The functions *S(t)*, *E(t)*, *I(t)*, *R(t)* describe the number of susceptible, exposed, infectious and removed (dead or recovered) people at time *t*. The total population *N* = *S(t)* + *E(t)* + *I(t)* + *R(t)* is assumed to be constant. However, an additional extra class is introduced. *P(t)* expresses the public perception of risk depending on the number of reported confirmed cases that are infectious, in severe/critical condition or dead. Furthermore, the demography^31^ of Turkey and the course of the disease for different age groups is considered (Bock W, Adamik B, Bawiec M, et al. Mitigation and herd immunity strategy for COVID-19 is likely to fail. 2020. https://doi.org/10.1101/2020.03.25.20043109). These data are summarized in Table 1.

**TABLE 1:** Demography of Turkey on December 31, 2019, and Severity of Symptoms

	Age Groups					
	0-39	40-49	50-59	60-69	70-79	80+
Asymptotic or mild symptomps	85.1 %	84.8 %	83.2 %	79.3 %	71.5 %	59.6 %
Severe symptomps	14.4 %	14.4 %	14.1 %	13.4 %	12.1 %	10.1 %
Critical symptomps	0.4 %	0.8 %	2.7 %	7.3 %	16.3 %	30.2 %
Proportion in the Turkish population	61.9 %	13.8 %	10.9 %	7.5 %	4.0 %	1.8 %

Several scenarios are presented in this study. The 4 scenarios serve to introduce the model and show the influence of the various parameters considered. First, results for a constant transmission rate are presented. Second, the influence of the average temperature and then the influence of human reactions in the population in combination with the temperature is presented. An exemplary government response is also considered.

The modeling starts at *t* = 1 day corresponding to March 11, 2020, which is the date of the first confirmed COVID-19 case in Turkey and ends at August 31, 2020, for demonstration purposes in the first 3 scenarios. The transmission rate in the basic form of SEIR models is typically taken as a constant. However, *β(t)* in Equation (1) is taken as transmission rate in this study. Temperature *T(t)* effects described by ξ ≥ 0, which is the strength of the response of transmission rate to temperature variations, government actions like school closures encoded in **α(t)** and individual reactions given by the perception of risk *P(t)*, which decreases when less people die and increases when people die more often due to COVID-19, as well as the response strength κ ≥ 0 are included in Equation (1)(1)
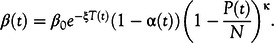



The codomain of *α(t)* is [0, 1] while it is [0, *N*] for *P(t)*. Thus, increasing government actions **α(t)** in the second factor in Equation (2) or increasing risk perception *P(t)* in the final factor in Equation (2) lead to a decreasing transmission rate. The proposed compartmental model is given in Equation (2) by(2)
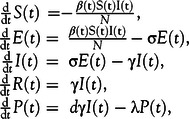
where σ^−1^ is the mean latent period, γ^−1^ the mean infectious period, *d* the proportion of severe and critical cases, λ^−1^ the mean duration of public reaction and β_0_ the original transmission rate. In addition to the temperature effect ξ, the humidity should also play a role as seasonal reality. However, to the best of my knowledge, there is no peer-reviewed quantitative information about this influence on August 04, 2020. Note that the information ξ_h_ on the strength of the response of transmission rate to humidity variations would lead to an additional factor 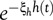 in Equation (1). In the framework of this study, the temperature is strongly correlated to the humidity. Hence, climatic effects are modeled with the temperature. The average temperature distribution of the months between March and June in Turkey are taken for the year 2020. The average temperatures of the months between July and December are taken for the year 2019.^32^ These data are summarized in Table 2. In this way, an attempt is made to make the most accurate analysis possible for the months in 2020. All the accessible data are given in the literature.^33-37^ The modeling parameters are summarized in Table 3.

**TABLE 2:** Average Temperatures of Certain Months in 2019 and 2020

Month	Average Temperature [°C]
March 2020	9.5
April 2020	12.1
May 2020	17.6
June 2020	21.7
July 2019	24.5
August 2019	25.3
September 2019	21.2
October 2019	17.4
November 2019	11.5
December 2019	6.5

**TABLE 3:** Parameters of the Model in Equation (2)

Parameter	Value
σ^−1^	3 days
σ^−1^	5 days
λ^−1^	11.2 days
β_0_	0.56
ξ	0 or 0.0383 K^-1^
κ	0 or 2254.1
α_0_	0 or 0.13
*S*(1)	83154997
*E*(1)	0
*I*(1)	1
*R*(1)	0
*P*(1)	0

Note that the response strength κ has been chosen as highest comparable value found in a previous publication, where the Spanish flu was analyzed,^30^ which means that the population is exceptionally careful and very well informed, while α_0_ is taken as low as possible due to the harsh social economic results of any action the government takes. Moreover, values zero for ξ, κ, and α_0_ are unrealistic and serve demonstrations such that it is possible to see the outcome of no reaction of the population as well as the government. The individual response of the population in Equation (1) is increasing with increasing number of infectious people. However, the response of the government *α(t)* is taken as a varying function due to published evidence.^38^ It is assumed that the government can take action for 2 mo continuously. The measures are then relaxed for 1 mo and used again for 2 mo. In total, the response of the government is given by(3)
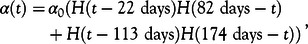
where *H(t)* is the Heaviside step function. Day 22 corresponds to April 01, 2020, 82 to May 31, 2020, 113 to July 01, 2020, and 174 to August 31, 2020.

Equation (2) is rewritten in Equation (4) as(4)
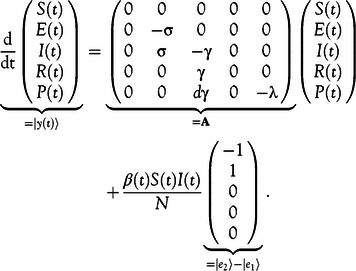



Equation (4) is solved by use of the classic fourth-order Runge-Kutta method.

### Review of the Runge-Kutta Method

Runge-Kutta methods are widely used in different fields like mathematical physics and engineering for solving ordinary differential equations numerically. For this purpose, the first-order initial-value problem in Equation (5) is considered (5)
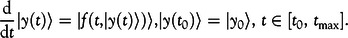



Dividing the interval [*t*_0_, *t*_max_] into *M* subinterbals [*t*_*m*_, *t*_*m*+1_] with *m* = 0,1, …, *M* − 1, *t_M_* = *t*_max_ and *h* = *t*_*m*+1_ − *t_m_*, integrating Equation (5) over each subinterval, using the mean value theorem, defining 
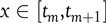
 and approximating 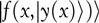 by a linear combination of the vectors 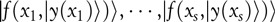 leads to Equation (6) (6)
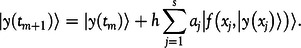



By choosing different values for the parameters *s*, *a_j_* and *x_j_* different Runge-Kutta formulations can be obtained. The most widely used form, the classic fourth-order Runge-Kutta method, is obtained for *s* = 4 in Equation (7)(7)
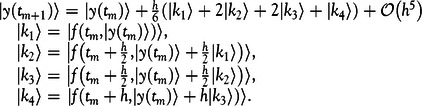



Hence, 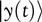 is determined numerically by iteration.

### Application to the Modified SEIR Model

The initial-value problem of our modified SEIR model in Equation (4) can be written as Equation (8)(8)
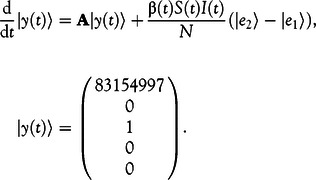



Thus, it is of the same form as Equation (5) and the classic fourth-order Runge-Kutta method can be used. Note that the step size can be chosen freely as time steps, such as days or hours, while smaller steps increase the accuracy of the numerical calculation. However, the computation time also increases with decreasing step sizes.

## RESULTS

### Demonstration of Exemplary Results of the Modified SEIR Model

**FIGURE 1:**
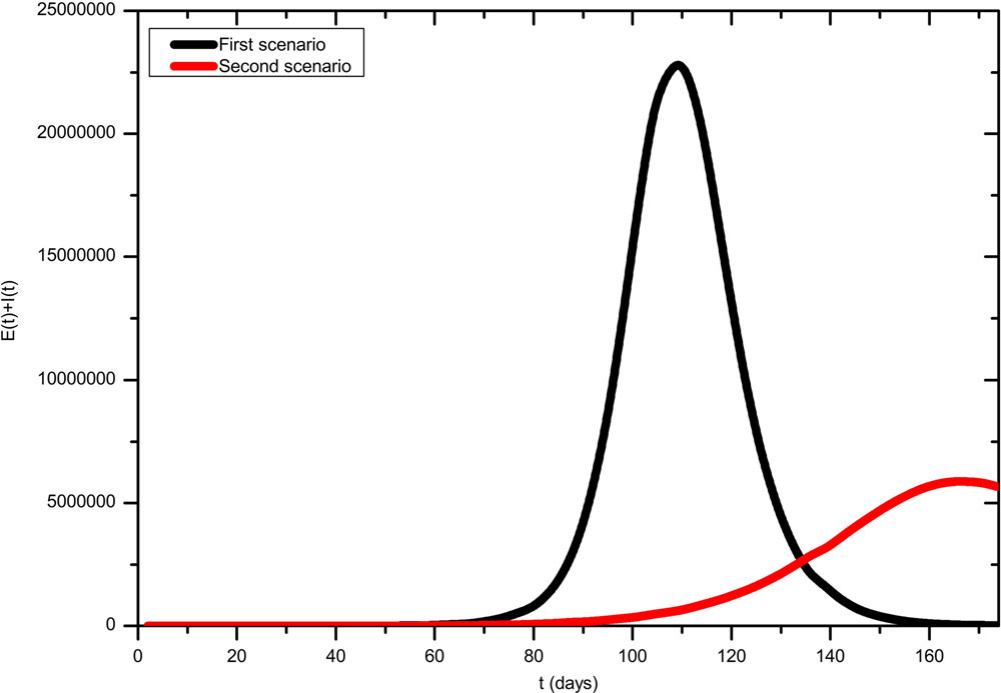
Model Simulation for the Development of the Outbreak Until August 31, 2020 Without and With Seasonal Effects While Individual and Government Actions Are Neglected.

**FIGURE 2:**
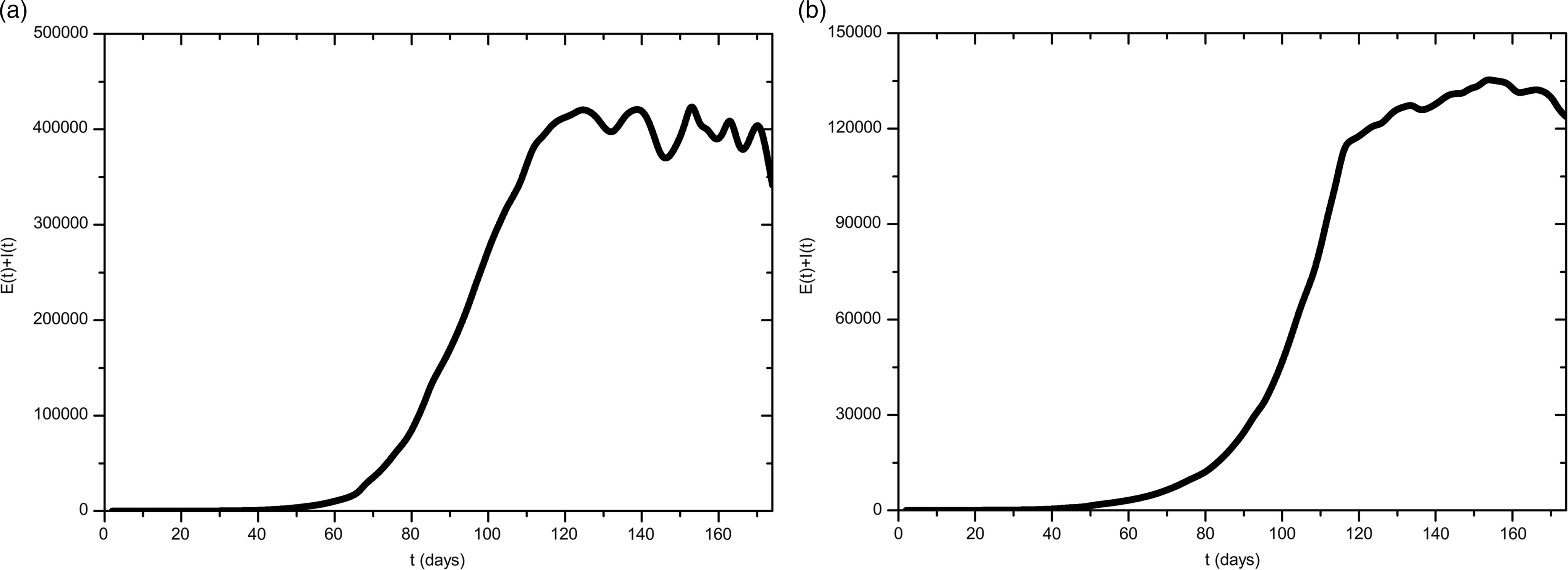
Model Simulation for the Development of the Outbreak Until August 31, 2020, (a) With Individual Action and (b) With Individual as Well as Government Action.

A total of 4 scenarios are presented graphically. The first scenario in Figure 1 is naive and unrealistic, because it assumes both constant temperature and no response from the population as well as the government, ie, ξ = α_0_ = κ = 0. The second scenario shows a projection that takes seasonal effects of Turkey into account, ie, just α_0_ = κ = 0. Moreover, Figure 2a shows the result for just α_0_ = 0 and Figure 2b takes all reactions and the weather into account.

### Comparison of the Numerical Model With the Real Data of Turkey

**FIGURE 3:**
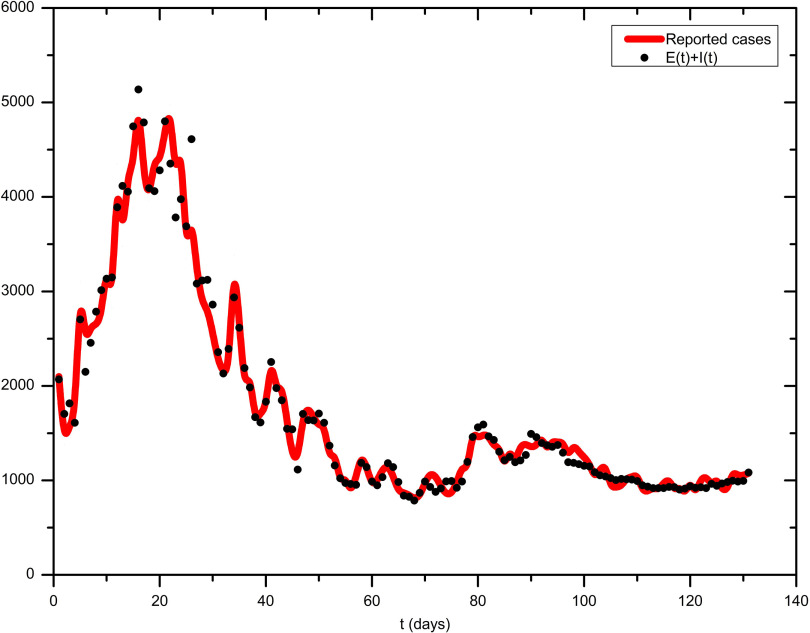
Fit of the Presented Model in Equation (4) to the Real Data of Turkey Between March 27, 2020, and August 04, 2020.

Conventional fitting to reported real data^39^ of infected coronavirus cases in Turkey with the presented model needs to be done carefully. Although the first case was confirmed on March 11, 2020, the daily cases were not published until March 27, 2020. Furthermore, note that data analyses with confirmed cases should be done carefully due to the delay of 1 to 2 wk between the number of confirmed cases calculated in this study and official reports. Moreover, there were a large number of different government responses that were put into effect at different times and are still partially valid. However, studies^40^ have shown that these can be summarized and there were certain days when the measures became stronger (on January 24, 2020; February 05, 2020; February 07, 2020; March 09, 2020; March 16, 2020; March 18, 2020; March 21, 2020; March 24, 2020; March 27, 2020; March 28, 2020; April 11, 2020; April 18, 2020) or weaker (on April 13, 2020; April 20, 2020; June 01, 2020; July 22, 2020). Hence, the government response function *α(t)* is assumed to be given by Equation (9)(9)
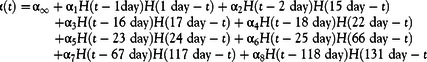
during the fitting process. The coefficients are determined by the fit and illustrate the full impact of government actions at the specified time. Furthermore, the coefficients are bound by the constraints α_2_ = α_4_ = α_6_ and α_3_ = α_5_. Due to some permanent measures, an additional coefficient α_∞_ is also considered. The result of the fit is depicted in Figure 3. The coefficients and the response strength κ of the risk perception among the population are found in Equation (10) to be(10)
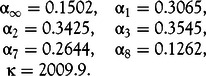



## DISCUSSION

The number of infections in both scenarios of Figure 1 shows that the situation can be extremely serious. Overall, an extremely large number of people would be affected (in these unrealistic cases). However, rising temperatures lead to a smaller number of exposed and infectious people. Moreover, the peak shifts from day 110 to day 167, ie, from June 28, 2020, to August 24, 2020. In the third scenario in Figure 2a only an individual but strong reaction is considered. The peak value at August 10, 2020, as well as the total number of all cases is significantly lower in comparison to the previous situations. Furthermore, fluctuations can be seen from July 12, 2020, onward. These arise because the individual response decreases as the numbers decrease, causing them to start rise again. The behavior of the curve shows that the number of diseases is still high. Even a well-prepared health-care system with a relatively young population (like in Turkey) could collapse. Because the highest published value was chosen for the individual response κ, it is obvious that a government response α(*t*) ≠ 0 (which has already occurred) is inevitable. However, it should be noted that a strong and long-term government response would lead to the suppression of the epidemic in Turkey. This method has certain disadvantages: On one hand, social and economic risks have to be considered. On the other hand, this would not result in herd immunity (Bao L, Deng W, Gao H, et al. Reinfection could not occur in SARS-CoV-2 infected rhesus macaques. 2020 and Wu F, Wang A, Liu M, et al. Neutralizing antibody responses to SARS-CoV-2 in a COVID-19 recovered patient cohort and their implications. 2020).

Additionally, the borders would have to be strictly controlled for a long time, because even a single super-spreader would condemn the strategy to failure. For these reasons, a different approach is modeled here. The smallest α_0_ that has been published is assumed. The government intervenes with this strength for a total of 2 mo to then loosen the measures by 1 mo. This procedure is continued periodically until vaccination and medication are approved by the relevant authorities. Results are depicted in Figure 2b. The highest point is observed for August 10, 2020. The fluctuations due to the individual response and the maximum have decreased significantly. The curve also shows when the government is not intervening. Between day 83 and day 112, the curve increases exponentially.

The purpose of this study is to propose a model with which different intervention scenarios can be considered. Hence, an analysis of the sensitivity with regard to the individual response strength κ and the government response strength α_0_ was carried out in the numerical experiments. As expected, a decreasing κ can be compensated for with increasing α_0_ (and vice versa). Values around κ = 110 and α_0_ = 0.9 lead to suppression while the scenario in Figure 2b leads to a mitigation strategy. However, even smaller government response strengths α_0_ lead to high numbers of infections that could yield a collapsing health-care system.

The comparison of the model with the existing real data of coronavirus cases for Turkey is depicted in Figure 3. Remarkably, a good agreement between the model and the real data can be observed. The intervention of the government modeled by *α(t)* in this case is much more involved than in the demonstrations and includes 9 parameters. Equations (10) show that the government intervened much more strongly and for a longer period of time compared with the exemplary scenario presented in Figure 2b. In addition, the early fluctuations in the curves show a strong response κ from the population. This leads to small fluctuations of the theoretical results around the real data at the end of the fit. This could possibly be improved by a time-dependent κ(*t*). However, it is quite difficult to make well-founded assumptions about the course of such a function.

It should be noted that the quantitative data determined by fitting do not provide any information about which specific reaction by the government or the population is reducing the spread of the virus to what extent, as many reactions were taken in parallel. It is, therefore, only possible to evaluate the measures as packages that were valid in certain periods of time and lead to the results in Equation (10).

## CONCLUSIONS

In this study a modified SEIR model is presented. The advantage of the proposed model is the inclusion of the influencing factors weather and individual as well as government response. Moreover, the proposed model is quite simple. Parameter estimates from previously published articles that analyze the Spanish flu were used for demonstrating the results of the model without classical fitting. The approximative values for the individual and government responses were obtained from the 1918-1920 Spanish flu outbreak due to certain similarities of both pandemics. Moreover, well-known epidemiologic parameters for COVID-19 are used. As last demonstration, the model is used for an exemplary proposition of a mitigation strategy, whereby the population is quite cautious in comparison to the 1918-1920 Spanish flu outbreak. However, the scenario gives no indication of how such a strong reaction can be evoked. For example it could be realized by informing the population for months by public-broadcasting in a science-based manner, calling for caution and providing surgical masks as well as other necessary materials.

Additionally, the government could explain the strategy regularly to show how important the individual response is. Above all, it could explain that careless individual response naturally leads to a stronger government reaction, which in turn would trigger major social and economic upheavals among the population. Furthermore, the scenario shows that even a strong response from the population is not enough and the entire transfer of the responsibility to the population is not a viable path. The government has to at least react periodically to mitigate the outbreak. It should be noted, however, that within the framework of this study even a weak periodic intervention in combination with a strong individual reaction leads to the mitigation of the outbreak.

In addition to the presented scenarios, which serve as a demonstration of the model, the model was also fitted to the existing real data for Turkey. A good agreement of the model with these data was found, which shows that this method can, in principle, serve to quantify individual human reactions and government decisions. However, this is always possible for total reactions valid at certain periods of time. The result of the model shows that there was a strong individual reaction at the beginning of the outbreak. However, this seems to have weakened over time. The results also show that the government intervened heavily between the second and 66th day. However, the measures were subsequently weakened in total. In addition, permanent measures taken by the government are recognizable by α_∞_. In any case, it can be seen from the fit and the scenario of Figure 2b that the pandemic outbreak could be kept under control in certain circumstances by consciously controlling the individual reaction and coordinated as well as possibly only relatively weak interventions by the government.

The framework of this model should be considered as a first step. Obviously, it is open to further improvement with other effects like humidity. Additionally, the analysis performed in this study should be done for all provinces of Turkey due to great differences in the average temperatures (for example, in Ardahan and Mardin) and level of education. Moreover, it should be noted that the analysis presented here can be carried out for all provinces around the world. Due to the local differences, such a procedure seems to make sense, because it can be used to plan local measures. This is of particular value because many countries are currently pursuing various strategies. Due to the distribution of the virus within the first wave around the world, it is possible that a potential second wave could consist of many different local outbreaks in every country.

## Supplementary material

To view supplementary material for this article, please visit https://doi.org/10.1017/dmp.2020.322.
